# Seven Strategies Implemented in Response to the 16th Ebola Virus Disease Outbreak in the Democratic Republic of Congo: Lessons Learned over a Three-Month Period

**DOI:** 10.3390/v18010028

**Published:** 2025-12-24

**Authors:** Dieudonné K. Mwamba, Karl B. Angendu, Waly Diouf, Marie-Claire Mikobi, Olive Leonard, Danny Kalala, Nella Ntumba, Deogratias Kakule, David K. Kayembe, Emilia Sana, Bienvenu Kabasele, Jack Katya, Alice Montoyo, Béatrice Serra, Henriette Bulambo, John Otshudiema, Serge Kapanga, Olea Balayulu, Jeanpie Muya, Erick Kamangu, Richard Kitenge, Gaston Tshapenda, Cris Kasita, Mory Keita, Francis K. Kabasubabo, John Kombe, Mathias Mossoko, Christian B. Ngandu, Célestin Manianga, Gregory Moullec, Christina Zarowsky, Pierre Z. Akilimali

**Affiliations:** 1 Ebola Incident Management System, Democratic Republic of Congo National Public Health Institute, Kinshasa P.O. Box 3243, Democratic Republic of the Congo; dieudonne.mwamba@insp.cd (D.K.M.); dr.dioufwaly@gmail.com (W.D.); marieclairem9@gmail.com (M.-C.M.); olivia.leonard@who.int (O.L.); danny.kalala@insp.cd (D.K.); nellantumba41@gmail.com (N.N.); kakuled@who.int (D.K.); david.kayembe@insp.cd (D.K.K.); emilia.sana@insp.cd (E.S.); b.kabasele@insp.cd (B.K.); katyaj@who.int (J.K.); alice.montoyo@coral.alima.ngo (A.M.); beatrice.serra@coral.alima.ngo (B.S.); ywt6@cdc.gov (H.B.); otshudiemaj@who.int (J.O.); sergekapanga@gmail.com (S.K.); oleabalayulu@gmail.com (O.B.); jeanpie.muyawetu@insp.cd (J.M.); erick.kamangu@insp.cd (E.K.); richard.kitenge@insp.cd (R.K.); tshapindon86@gmail.com (G.T.); snabcris@gmail.com (C.K.); mokeita@who.int (M.K.); or fkabasu13@gmail.com (F.K.K.); johnkombe171@gmail.com (J.K.); mathias.mossoko@insp.cd (M.M.); christian.ngandu@insp.cd (C.B.N.); cmanianga@gmail.com (C.M.); pierre.akilimali@unikin.ac.cd (P.Z.A.); 2 Department of Social and Preventive Medicine, School of Public Health, University of Montreal, Montreal, QC H3T 1J4, Canada; gregory.mollec@umontreal.ca (G.M.); christina.zarowsky@umontreal.ca (C.Z.); 3 Department of Anthropology, Kinshasa School of Public Health, University of Kinshasa, Kinshasa P.O. Box 127, Democratic Republic of the Congo; 4 Inserm U1094, IRD UMR270, Univ. Limoges, CHU Limoges, EpiMaCT—Epidemiology of Chronic Diseases in Tropical Zone, Institute of Epidemiology and Tropical Neurology, OmegaHealth, 87000 Limoges, France; 5 Laboratory of Sociology, Anthropology, and Psychology, Cheick Anta Diop University, Dakar 10700, Senegal; 6 World Health Organization, 1211 Geneva, Switzerland; 7 The Alliance for International Medical Action, 75000 Paris, France; 8 United States Centers for Disease Control and Prevention, Atlanta, GA 30329, USA

**Keywords:** Ebola outbreak, incident management system, Public Health Emergency Operations Center, National Public Health Institute, Democratic Republic of Congo

## Abstract

The 2025 Ebola outbreak that ravaged the Bulape Health District (HD) in Kasai, Democratic Republic of Congo (DRC), was tackled using the incident management system (IMS) model. The Bulape HD is located in the Mweka territory, which has experienced two Ebola epidemics: one in 2007 and another in 2008. The IMS comprises seven strategies recommended for an effective response to an Ebola outbreak: (i) thorough investigation, (ii) strengthening infection prevention and control measures in the community, (iii) ensuring that medical care is provided by experienced professionals, (iv) strengthening risk communication and community engagement (RCCE), (v) ring vaccination, (vi) operational research, and (vii) anchoring interventions in the existing health system. We share our experience implementing these seven strategies and compare them with those utilized during three previous Ebola outbreaks. This paper describes our achievements, the resulting benefits, and the factors that facilitated the implementation of the aforementioned strategies. A literature review and interviews were conducted. The atlas.ti 22 software was used for data analysis. Implementing these seven strategies contributed to an effective response, largely due to the experience and expertise of those involved but also thanks to the support of technical and financial partners (TFPs) and the engagement of the local community. Challenges such as geographical accessibility, the fragile health system, the community’s strong attachment to traditional practices, and negative reactions to healthcare—which was widely discredited, with many of those involved expressing a lack of faith in its effectiveness—were major obstacles. To overcome these challenges, an integrated approach was utilized, combining a rapid comprehensive response with deep and respectful community engagement. The support and alignment of TFPs were invaluable during this process. The RCCE pillar proved key to successful IMS implementation. Our experiences will be useful during the next Ebola outbreak in the DRC; additionally, they may also help to inform the response to similar outbreaks in other countries.

## 1. Introduction

Ebola virus, member of genus Ebolavirus, family Filoviridae, have a non-segmented, single-stranded RNA that contains seven genes: (a) nucleoprotein, (b) viral protein 35 (VP35), (c) VP40, (d) glycoprotein (GP), (e) VP30, (f) VP24, and (g) RNA polymerase (L). All genes encode for one protein each, except GP, producing three pre-proteins due to the transcriptional editing. These pre-proteins are translated into four products, namely: (a) soluble secreted glycoprotein (sGP), (b) Δ-peptide, (c) full-length transmembrane spike glycoprotein, and (d) soluble small secreted glycoprotein. Further, shed GP is released from infected cells due to cleavage of GP by tumor necrosis factor α-converting enzyme. There are five distinct species of Ebola virus known to infect humans: (i) Reston, which is asymptomatic in humans and not lethal, (ii) Sudan, which has a human fatality rate of approximately 50%, (iii) Tai Forest, which is almost non-lethal, (iv) Bundibugyo, with a human fatality rate between 25 and 50%, and (v) Zaire, the most lethal and most responsible for Ebola virus disease (EVD) in the Democratic Republic of Congo (DRC), with a human fatality rate ranging from 60 to 90% [1,2,3,4,5]. Of the five species of Ebolavirus, only the last four have been responsible for outbreaks in sub-Saharan Africa (SSA) [5]. Vaccines have been and continue to be developed to combat the different species of Ebola virus. rVSV-ZEBOV (Ervebo) and Ad26/MVA (Zab-deno/Mvabea) have been tested and provide almost 100% protection against the Zaire species [6]. They are the only ones approved for use in humans. Other vaccines against other species of Ebola virus are either in development, undergoing trials, or not yet approved by the international authorities responsible for doing so [7].

Ervebo^®^ is the one that has been used during recent outbreaks in the DRC, applying the ring vaccination strategy: vaccination of close contacts and potential contacts of a confirmed or probable case. Once Ervebo^®^ is injected, the recombinant virus enters certain cells in the body and produces the Ebola virus GP. The immune system recognizes this protein as foreign and mounts a robust response, producing specific antibodies and activating memory T cells. If the person is then exposed to the real Ebola Zaire virus, their immune system is ready to neutralize it quickly [8,9].

Ebola virus disease can be transmitted between animals through infected bodily fluids or objects contaminated with these fluids. Animals can also transmit the disease to humans, particularly through the consumption of contaminated meat or the handling of infected animals. Humans can also infect each other through blood or other biological fluids, objects contaminated with bodily fluids from a person who is sick or has died from EVD, or the semen of a person who has recovered [5].The genetic conservation of the Ebola virus, its true origin, its natural reservoirs, and the dynamics of its transmission from wildlife to humans remain unclear, complicating the prediction and prevention of outbreaks and/or re-emergence in the DRC. EVD mainly occurs in SSA. The first cases imported into Europe and the United States were recorded in 2014 [10,11,12]. The longest and deadliest Ebola outbreak struck the western part of SSA in the same year, mainly affecting Guinea, Liberia, and Sierra Leone. It resulted in 28,616 cases and 11,310 deaths, with a case fatality rate of 39.5% [10,13]. Since its initial detection in 1976, the virus has not undergone any major selective mutations [10,14]. The 2021 outbreak in Guinea was linked to a lineage closely related to previous strains, suggesting a potential re-emergence following latent infection rather than a new mutation [15]. However, one study suggests that the Ebola virus has undergone significant mutations since it was originally discovered, particularly in the glycoprotein during the 2014 outbreak, indicating genetic diversity and adaptation despite strong purifying selection [16]. Recent genomic surveillance has identified variants, such as the Makona variant from the 2013–2016 outbreak, with unique pathogenic characteristics [17]. This offers an ongoing opportunity for sustainable investment and development of vaccines, treatments, and diagnostics.

However, successful implementation of such biomedical interventions requires comprehensive public health and One Health approaches. One of the main lessons learned from the Ebola outbreak in Africa is that, in addition to weaknesses in health systems, slow and unpredictable funding contributed significantly to the slow response. The DRC is currently experiencing its 16th Ebola outbreak, while the Kasai region specifically is in the midst of its third outbreak, the latest of which struck the Bulape Health District (HD) in 2025. It has emerged alongside other outbreaks and major public health events in the DRC, including instances of Mpox, cholera, and measles, as well as malnutrition in several regions [18].

The combined efforts of the public authorities, technical and financial partners (TFPs), and community mobilization enabled the early detection of the outbreak, prompt implementation of response measures, and control of the contamination chain. This allowed us to contain the outbreak within Bulape and prevent it from spreading to neighboring districts or regions. The outbreak also did not last as long as other Ebola outbreaks in the Kasai region: the first, in 2007, lasted 230 days; the second, which took place in 2008, lasted only 88 days, and the same duration was reported for the third outbreak [18,19]. The outbreak in the Bulape HD was mainly rural, with 53 people confirmed to be infected, of whom 34 died, representing a case fatality rate of 64.2%, compared to 71% during the 2007 outbreak and 44% in 2008 [18].

Since 2023, with the operationalization of the Public Health Emergency Operations Center within the National Public Health Institute, the DRC has undergone a paradigm shift in the management of outbreaks and other public health emergencies (PHEs), adopting an incident management system (IMS) model as a successor to the previous system, which was not standardized. The Ebola outbreak in Bulape in the Kasai region was one of the first incidents in which this new approach was implanted in response to PHEs such as Mpox and cholera that are still ongoing in the DRC. This article documents the practices used during this innovative management of the Ebola outbreak and compares them with previously used strategies based on the three recent Ebola outbreaks that occurred in the provinces of North Kivu (13th and 15th outbreaks) and Equateur (14th outbreak). Our aim is to critically analyze the IMS’s added value, its limitations, and the specific challenges and lessons learned during the response.

## 2. Methodology

### 2.1. Study Design

A qualitative methodology and document analysis were used to examine the seven recommended strategies [20]: thorough retrospective and prospective investigation; strengthening infection prevention and control (IPC) measures in the community; holistic case management provided by experienced staff; strengthening risk communication and community engagement (RCCE); ring vaccination; operational research; and anchoring interventions in the system.

### 2.2. Sources

The analysis was based on a set of three reports on the last 3 Ebola outbreaks in the DRC, which occurred in the provinces of North Kivu (during the 13th and 15th outbreaks in 2021 and 2022) and Equateur (the location of the 14th outbreak in 2022); two reports on the last two Ebola outbreaks in the Kasai region; reports from the ministry of health, the World Health Organization (WHO) and other partners; as well as various documents related to the most recent Ebola outbreak. They describe the responses to outbreaks that occurred between 2007 and 2025 and the seven recommended strategies that will facilitate a better response, comparing the situation prior to the most recent Ebola outbreak with observations made during the outbreak.

### 2.3. Analysis Framework and Complementary Qualitative Data

To guide the investigation, a qualitative analysis grid was designed. Each report was described according to whether or not the seven recommended strategies had been implemented. In addition to the analysis, individual interviews (*n* = 31) were conducted with operational managers from the pillars and sections, as well as with central- and local-level managers and community leaders involved in the response. The data for the qualitative part was collected over a two-month period between October and November 2025. The investigators were experts from the Ministry of Public Health and the ministry’s technical and financial partners, with the support of local experts. They were trained in interview techniques and qualitative data collection. Supervisors were also assigned to ensure the quality of the data and information. They were trained for this purpose. The training was provided by university professors, who also validated the analysis results. They were experts in various fields: anthropology, human medicine, epidemiology, sociology, veterinary medicine, biology, and biostatistics. Triangulation was performed using data from the literature review and qualitative methods. atlas.ti 22 was used for data analysis.

## 3. Results

### 3.1. Responses to the 3 Previous Ebola Outbreaks vs. The 16th Outbreak

The response to the last three Ebola outbreaks and the one in Bulape is summarized in Table 1. The strategies were identified and selected based on the components of the IMS and the guidelines outlined in a recently published article [10,19,21,22]. They were based primarily on the seven effective response strategies recommended in a recent study on the 16th Ebola outbreak in the Bulape health district [20].

### 3.2. Achievements, Benefits, and Factors That Enabled the Successful Response to the 16th Outbreak

Firstly, surveillance was deemed to be particularly effective. This was marked by a steady increase in the number of alerts reported and the high proportion of subsequent investigations. In addition to the expertise of the surveillance pillar members, these positive results were largely attributed to the large-scale deployment of field epidemiologists and an improved community alert system via the members of the RCCE pillar. This made it possible to break the chain of human-to-human transmission. In Liberia, similar results were achieved after involving field epidemiologists [23].

Epidemiological surveillance activities were also facilitated by isolating the epicenter of the outbreak and through the collaborative attitudes of communities and specific cultural practices relating to the management of the mourning period. More specifically, in community groups in the Bulape health district, observing widowhood practices involved a period of self-isolation, which provided an opportunity for contact tracing and monitoring. This not only facilitated availability and easy access for teams, but also limited the mobility of contact cases, thereby breaking the chains of transmission. Finally, the mobilization of local health system actors made it possible to engage legitimate actors who were able to interact with communities. This highlights the added value of integrating response interventions into the health system.

Secondly, IPC measures were quickly implemented in areas where they were needed. This helped to quickly break the transmission chain and protect high-risk groups such as health workers. This was made possible by the rapid deployment of IPC experts and the support of TFPs. This approach is strongly recommended by the World Health Organization [24].

Thirdly, with regard to the case management pillar of the IMS, Ebola Treatment Centers (ETCs) were set up based on the recommended protocols, with clearly separated areas (red and green zones) and a transit center to support the ETCs. This improved staff safety and reduced the number of nosocomial infections. The digitization of care activities has improved response times. This has been made possible by the joint efforts and support of TFPs, through the transfer of skills and technologies.

(a) Fourthly, as a crosscutting pillar, RCCE has been the backbone of IMS innovation in the 16th EVD epidemic in the DRC, supporting all seven pillars from the generation of increased surveillance alerts to motivating community participation in the IPC, case management, vaccination efforts, and research, and even supporting overall coordination.
(b) RCCE’s support for IM and coordination: In mid-September 2025, the community was in turmoil, with several deaths and few successful recoveries among hospitalized patients. This resulted in increased resistance to case management uptake. There is a strong shared belief in traditional authorities and values in the Bulape community. Through mobilizing traditional leaders (heads of clans), the RCCE supported IMS coordination. The RCCE pillar facilitated a tacit deal, also called “the pact”, between the response coordination and the head of clans within the villages, who made a verbal commitment to work together to stop the spread of EVD within 6 weeks. The pact included agreed-upon mutual commitments and consideration of traditional leaders’ concerns during the response coordination. The leaders agreed to sensitize and motivate community members to facilitate engagement and improve health-seeking behavior through increased community alerts, identification of contacts, as well as greater Ebola vaccine demand. The response coordination commitment included improvement of the quality of case management, i.e., free treatment of other diseases and improved nutrition conditions within Ebola treatment and transit centers, taking into account the culturally accepted diet. Coincidentally, only a week after the agreement was established, the last case of EVD was discharged from the Ebola treatment center on September 25th. These practices also proved effective during the outbreak in West Africa [25].
(c) RCCE supported both the surveillance and case management pillars, respectively, mainly through the increased community alerts and progressive healthcare uptake. Also, through community-based surveillance, the RCCE collaborated with the surveillance pillar’s scaling up of the training of Community health workers. Community engagement activities have promoted transparent communication about the care offered by ETCs, which has increased patients’ trust in and acceptance of health services.
(d) Support for the vaccination pillar: The 16th EVD response has been recorded as one of the best, if not the best, with regard to ERVEBO vaccination uptake. The increased demand for vaccination uptake in Bulape health district is clearly the result of the RCCE sensitization efforts. In fact, the vaccination strategy shifted its focus from frontline workers to contacts (and contacts of contacts) in a geographical strategy, thanks to combined RCCE approaches that involved promoting the benefits of vaccination. Public awareness was raised through infodemic management strategies involving collaboration with experts from other domains; responding to community feedback and rumors was critical, and clear information about vaccines (side effects, efficacy, safety, etc.) was provided to gain the public’s trust.
(e) RCCE support for IPC: As with surveillance, the presence of RCCE in the community supported the IPC in simplifying technical prevention messages to improve the uptake of public health measures and apply the recommended IPC measures, including safe and dignified burials.
(f) The research pillar of the IMS has also been directly supported by the RCCE through the implementation of two distinctive Rapid Analysis Quality Surveys, at the beginning and towards the end of the response, and indirectly through supporting rapid assessments and studies within the community.

Overall, the RCCE within the IMS 16th EVD National Response plan of action highlighted five distinctive strategic axes: (i) coordination among RCCE pillar actors, (ii) promotion of preventive measures, (iii) demand generation, and (iv) management of infodemics, monitoring, and evaluation. Coordination among all RCCE key players minimized duplication and maximized resources throughout integrated action plans and daily coordination meetings. In addition, two impactful initiatives were established by the RCCE pillar to improve awareness and manage infodemics. In schools, “genie en herbe” competitions were held, focusing on EVD, which enabled students to be better informed about the disease. Therefore, students were leveraged as key RCCE actors within their respective families and their school communities.

In addition, empowering local actors, including community, religious, and associative leaders, as well as artists, journalists, and other recognized local figures, as a frontline team in social mobilization and community engagement strengthened the connection between the response and the community. Since these actors have mastered the local language and understand the social, cultural, and anthropological realities of the area, this approach promoted better local ownership of the actions undertaken and helped anticipate and prevent rumors and xenophobic resistance toward teams coming from outside.

Fifthly, in addition to vaccinating those in the outbreak zone, we targeted frontline human resources in at-risk areas, including health workers in private healthcare facilities, traditional healers, pastors, and motorcyclists in neighboring areas and areas which we anticipated would be affected. those involved in the response and those providing routine public transportation. This prevented the occurrence of EVD in people who were exposed to the disease. This was a good practice that was made possible by widespread community acceptance and adherence to the vaccination program. The effectiveness of this strategy was highlighted in a study conducted in the DRC [26].

Sixth, operational research proved to be indispensable. It effectively provided potential solutions to outstanding issues that required clarification based on evidence. In addition to the initial assessment of the pillars and sections, which identified operational challenges and suggested solutions, particular focus was placed on qualitative studies. As the population of the Bulape health district is very attached to its customs and traditions, scientific evidence was used to identify the practices and perceptions that would have an impact on the response in the local area, which included funeral rites, polygamy, and the predominance of female caregivers. The success of the research activities was made possible by a work plan based on operational needs identified by the various pillars of the response and certain TFPs, as well as the support of the TFPs and the availability of competent care providers. The benefit of this innovative approach was that it helped us to make decisions based on scientific evidence. This approach has been strongly recommended in other research studies [27].

Finally, there was good coordination between stakeholders and interventions, with at least two meetings held each day. This enabled close monitoring of the implementation of operational activities in the field, in collaboration with the health system, and the efficient use of resources. This was made possible, in particular, by the alignment of TFPs, with the positive impact of containing the outbreak in a single health district. All of this was facilitated by managing the Ebola outbreak under the IMS model, according to which each stakeholder had a clearly defined role.

### 3.3. Challenges During the Response and the Possible Solutions

In Bulape, as in other rural areas of the DRC, the Ebola outbreak response was not just a medical challenge. It was a race against time that faced an ecosystem of challenges, where logistical and geographical access issues exacerbated health concerns, which were themselves amplified by socio-cultural practices. Common issues included accessibility issues and a lack of basic infrastructure, such as a water supply system. Communication problems, a weak health system, denial of the disease, refusal to seek treatment, reluctance to accept safe burials, rumors and misinformation, language barriers, and illiteracy also posed significant issues. To overcome these challenges, actions were based on an integrated approach that combined a rapid comprehensive response with deep and respectful community engagement, all within an extremely challenging operational context.

## 4. Discussion

Our comparative analysis of responses to recent Ebola outbreaks in the DRC revealed a significant shift towards a more integrated, systemic, and community-based model during the 16th outbreak in Bulape. The success of this intervention, characterized by rapid containment within a single health district, seems to be largely attributable to the rigorous application of the IMS model and the central role assigned to RCCE. The case of Bulape is a prime example of how RCCE, which has traditionally been considered a supporting pillar, can become the integrating force of the entire response. Its role was not incidental but fundamental, actively supporting all other pillars. The “pact” established in collaboration with traditional clan leaders is a textbook case of community engagement. This approach, which created shared responsibility and mutual trust, was a decisive catalyst in breaking down the last pockets of resistance. It echoes the conclusions of one study conducted during the outbreak in West Africa, which highlights the effectiveness of interventions being directly correlated with their alignment with local authority structures and social logic [25]. By acting as a trusted intermediary, the RCCE made it possible to translate public health imperatives into socially acceptable actions.

Innovative management of rumors and infodemics through “budding engineer” skills is also worth highlighting. Instead of a top-down message, this strategy empowered young people, transforming them into active agents of RCCE within their families. This practice is in line with the principles of “peer education,” which has proven effective in promoting behavioral change in various public health contexts. The shift from a “silo” model to an IMS structure resolved chronic coordination issues. The emphasis on anchoring interventions in the existing system and accrediting stakeholders eliminated duplication and optimized resource use. This is consistent with the recommendations on health emergency governance, which emphasize the need for centralized coordination and seamless integration of international and national actors in order to facilitate a coherent response [28].

The explicit integration of the “animal care” sub-pillar and the “One Health” approach into case management represents a conceptual and practical development. It recognizes the role of animal reservoirs in the spread of Ebola and allows for more holistic surveillance. This approach is supported by a study conducted in central Africa, in which the authors argue that the prevention of zoonotic diseases such as Ebola requires intersectoral collaboration to control human–animal–environment interfaces [29]. The research pillar was not an isolated entity but an integrated service whose work directly informed operational decisions. The identification of specific cultural practices (mourning, polygamy, and the role of female caregivers) enabled the RCCE and other pillars to tailor their messages and interventions. This use of real-time qualitative evidence to refine the response is a model of best practice. It echoes another study’s calls for “embedded” anthropology in health emergencies, which can provide rapid contextual analysis to overcome socio-cultural barriers [30].

Bulape’s success cannot be attributed to a single intervention but rather to synergy between several factors. (i) Enhanced surveillance: The combination of field epidemiologists and increased community alerts allowed us to establish a rapid detection and response system, similar to strategies that proved successful in Liberia [23]. (ii) Facilitating cultural practices: Unexpectedly, practices such as the widowhood period, by creating natural isolation, facilitated contact tracing. This illustrates the importance of understanding local practices not only as obstacles, but also as potential resources. (iii) Targeted and accepted vaccination: Extending vaccination to key actors (healers, motorcyclists) in neighboring areas is a proactive prevention strategy. Its effectiveness, demonstrated by massive uptake, supports the results of studies on the important role that trust plays in vaccination coverage in the DRC [26]. Despite these innovations, the response had to contend with profound structural challenges, such as geographical isolation, lack of basic infrastructure (water sources), and a weak health system. These problems serve as a reminder that the response to epidemics remains dependent on fundamental social and economic determinants. The solution implemented—an integrated approach combining a rapid response and deep community engagement—is probably the most robust in such a context.

The next steps in the wake of the Bulape outbreak and the epidemiological situation in Kasai should involve a rapid transition from emergency response to an integrated program of recovery, health system strengthening, and preparedness by placing communities at the center of all actions. The key to success lies in sustained commitment from authorities and TFPs, even after media attention has faded. Four steps are proposed: 1. Consolidation and vigilance, to be implemented in the short term: (i) enhanced surveillance, (ii) effective follow-up of recovered patients, and (iii) screening for viral reservoirs. 2. Health system strengthening, to be implemented in the medium term: (i) resumption of essential health services, (ii) human resource development, and (iii) infrastructure and logistics. 3. Community engagement and maintaining trust (ongoing): (i) recognition and dialogue, (ii) combating the infodemic, and (iii) psychosocial support. 4. Preparedness and prevention, in the long term: (i) development of a preparedness plan, (ii) effective implementation of the “One Health” approach, and (iii) socioeconomic development.

## 5. Conclusions

Bulape’s health future must be built on numerous systems, from the successful emergency response managed by an IMS model with a structured and systematic application of the seven recommended strategies to resilience. The seven strategies described in this study were fully implemented during the Bulape outbreak, in contrast to the three previous outbreaks. The Ebola outbreak in Bulape was a brutal test, but one that provided valuable lessons. Its official end marks an undeniable operational success which can be attributed to the courage of healthcare workers, the involvement of communities, and the mobilization of TFPs. However, considering this victory as an endpoint would be a strategic mistake. Rather, it should serve as a decisive starting point. The true legacy of this crisis will not be measured by the weeks of intense struggle but by the lasting transformation it has inspired. The most critical phase—that of systemic strengthening—begins now. By investing in survivors, restoring confidence, rebuilding a more robust health system, and actively preparing for the next threat, authorities and their TFPs have a unique opportunity to build back better. The ultimate goal for Bulape and Kasai is not only to recover from Ebola, but to become regions where such an outbreak could never again cause the same amount of chaos. The focus should be on transforming this ordeal into a catalyst for a future where health is no longer a victim of crises, but the pillar of resilience and community development. Today’s vigilance is the price of tomorrow’s security.

## Figures and Tables

**Table 1:** Summary of the response between the last three Ebola outbreaks vs. the most recent one in Bulape, Kasai.

Strategies	EVD Management According to the IMS Model	Old EVD Management Model
Thorough retrospective and prospective investigation.	Involvement of all pillars * whose interventions were carried out in the community. This helped improve investigations.	Investigations performed solely by the oversight committee ^. Interventions were conducted in silos, which delayed investigations.
Strengthening infection prevention and control (IPC) measures in the community.	The interventions were conducted in cooperation with the political and administrative authorities, together with the security services. This helped to inspire community support.	Security services were not systematically involved in community interventions, leading to considerable resistance.
Holistic case management provided by experienced staff.	In addition to involving expert and experienced human resources, we established sub-pillars of blood transfusion and animal care and integrated them into holistic treatments, improving the quality of care. A One Health approach was considered. With regard to animal care, in addition to advising the community not to consume animals found dead or undercooked meat, veterinarians were called in to provide better care.	The animal care subcommittee was not established, and the blood transfusion subcommittee was not considered as such. This led to negligence in the control of reservoirs and vectors.
Strengthening risk communication and community engagement (RCCE).	Close collaboration between the RCCE, surveillance, and other pillars facilitated the prompt implementation of operational interventions, such as raising awareness among the community and enhancing community engagement from the local political administration and traditional and religious leaders, as well as other key local influencers such as village musicians. This helped to foster community buy-in.	The collaboration between the RCCE and other pillars was not as strong as during the last outbreak, and preventative measures taken in collaboration between the RCCE pillar and other pillars were not as effectively synchronized as during that recent outbreak.
Ring vaccination.	The availability of real-time data in the national health information database facilitated the monitoring and refocusing of interventions. This enabled the monitoring of the vaccination strategy.	Vaccination data was not stored in the national database, making it difficult to effectively monitor vaccination coverage with respect to targets.
Operational research.	Spontaneously provided clarification based on evidence, using quantitative, qualitative, and mixed approaches.	The research commission was not an integral part of the coordination of the response, structurally or systemically. Each commission had its own small unit, with only a limited ability to conduct advanced analyses. This delayed the implementation of the “action research” approach.
Anchoring interventions in the system.	Better coordination around the incident manager (IM) through pillar and section managers with the involvement of TFPs enabled alignment with the system and the elimination of duplicate responders and interventions. The implementation of an accreditation system prior to deployment in the field and effective monitoring of responders and interventions were deemed effective.	There was no accreditation system for human resources deployed in the field. This limited the effective monitoring of responders and interventions. The consequences were non-alignment with the system, duplication, and inefficient use of resources.

* An organized group of experts in a specific field in the IMS; ^ an organized group of experts in a specific field in management without the IMS approach.

## Data Availability

The collected data is available and can be shared anonymously.
